# Celastrol alleviates atopic dermatitis by regulating Ezrin‐mediated mitochondrial fission and fusion

**DOI:** 10.1111/jcmm.18375

**Published:** 2024-07-22

**Authors:** Dandan Wang, Shan Jin, Hanye Liu, Xinyi Song, Hongyu Jin, Yilan Song, Hongwei Zhao, Liangchang Li, Guanghai Yan

**Affiliations:** ^1^ Jilin Key Laboratory for Immune and Targeting Research on Common Allergic Diseases Yanbian University Yanji China; ^2^ Department of Anatomy, Histology and Embryology Yanbian University Medical College Yanji China; ^3^ Department of Dermatology Affiliated Hospital of Yanbian University Yanji China

**Keywords:** atopic dermatitis, celastrol, Drp1, ezrin, mitochondrial fission and fusion

## Abstract

Celastrol, a bioactive molecule extracted from the plant *Tripterygium wilfordii* Hook F., possesses anti‐inflammatory, anti‐obesity and anti‐tumour properties. Despite its efficacy in improving erythema and scaling in psoriatic mice, the specific therapeutic mechanism of celastrol in atopic dermatitis (AD) remains unknown. This study aims to examine the role and mechanism of celastrol in AD using TNF‐α‐stimulated HaCaT cells and DNCB‐induced Balb/c mice as in vitro and in vivo AD models, respectively. Celastrol was found to inhibit the increased epidermal thickness, reduce spleen and lymph node weights, attenuate inflammatory cell infiltration and mast cell degranulation and decrease thymic stromal lymphopoietin (TSLP) as well as various inflammatory factors (IL‐4, IL‐13, TNF‐α, IL‐5, IL‐31, IL‐33, IgE, TSLP, IL‐17, IL‐23, IL‐1β, CCL11 and CCL17) in AD mice. Additionally, celastrol inhibited Ezrin phosphorylation at Thr567, restored mitochondrial network structure, promoted translocation of Drp1 to the cytoplasm and reduced TNF‐α‐induced cellular reactive oxygen species (ROS), mitochondrial ROS (mtROS) and mitochondrial membrane potential (MMP) production. Interestingly, Mdivi‐1 (a mitochondrial fission inhibitor) and Ezrin‐specific siRNAs lowered inflammatory factor levels and restored mitochondrial reticular formation, as well as ROS, mtROS and MMP production. Co‐immunoprecipitation revealed that Ezrin interacted with Drp1. Knocking down Ezrin reduced mitochondrial fission protein Drp1 phosphorylation and Fis1 expression while increasing the expression of fusion proteins Mfn1 and Mfn2. The regulation of mitochondrial fission and fusion by Ezrin was confirmed. Overall, celastrol may alleviate AD by regulating Ezrin‐mediated mitochondrial fission and fusion, which may become a novel therapeutic reagent for alleviating AD.

## 1 INTRODUCTION

Atopic dermatitis (AD) is a chronic recurrent inflammatory skin disease characterised by skin lesions, itchy exfoliation and skin barrier dysfunction.^1^ The occurrence of AD is related to immune dysfunction and abnormal skin barrier function.^2^ The pathological manifestations of AD include the imbalances in Th1, Th2 and Treg cell immunity and the increased Th2 cytokines IL‐4 and IL‐13, which may ultimately alter Th1‐ and Th2‐mediated immune responses and IgE‐mediated hypersensitivity.^2^ The current commonly used treatment method for AD is the local use of steroids, including topical corticosteroids, topical calcineurin inhibitors, or a combination of both.^3^ These therapeutic drugs can alleviate AD symptoms, reduce inflammation and prevent recurrence; however, long‐term use of drugs may result in significant side effects.^4^

Mitochondria are key players in innate immune pathways and are important drivers of inflammation caused by aseptic injury. As cytoplasmic organelles, they can fuse, divide and move along the cytoskeleton, thus forming a mitochondrial network in response to the cellular energy state,^5^ and are also the main sites for reactive oxygen species (ROS) production.^6^ Mitochondrial dynamics, which are mediated by the GTPases, include the physiological processes of fusion and fission. Mitochondrial fission is the process of dividing a mitochondrion into two separate entities. Mitochondrial fusion involves two mitochondria colliding end‐to‐end, undergoing membrane fusion at the site or end‐to‐side of collision and finally merging into one entity or forming a ring structure within a single mitochondrion. The dynein‐related protein 1 (Drp1) and mitochondrial fission protein 1 (Fis1) can promote mitochondrial fission, and optic atrophy 1 (Opa1) and mitofusin (Mfn) can catalyse the fusion of mitochondrial inner and outer membranes, respectively.^7^ Drp1 is the central mediator of mitochondrial fission. When Ser616 of Drp‐1 is phosphorylated, the Drp‐1 is activated and recruited from the cytoplasm to the outer membrane of mitochondria, promoting mitochondrial rupture.^8^ When mitochondrial integrity is compromised, molecular patterns associated with mitochondrial damage bind to pattern recognition receptors and trigger inflammation.^9^ Some scholars have demonstrated that gene expression of Drp1 is increased in skin biopsies from AD patients.^10^ The imbalance in mitochondrial dynamics can affect the redistribution and maintenance of mitochondrial networks,^11^ which might lead to various diseases, including neurodegenerative diseases,^12^ liver ischemia–reperfusion injury,^13^ inflammatory airway diseases,^14^ and various cancers.^15^ However, the effect of mitochondrial dynamics abnormalities on AD has not been reported.

Ezrin is a major member of the Ezrin/radixin/moesin protein family,^16^ which plays the role of cross‐linking membranes and cytoskeleton.^17^ There are two forms of Ezrin, that is, inactive or active. The inactive Ezrin is located in the cytoplasm. Upon phosphorylation, active Ezrin translocates to the cell membrane.^18^ The tyrosine Thr567 phosphorylation site at the carboxyl end of Ezrin is the main target for its activation.^19^ Ezrin can induce Th0 to differentiate into Th2 cells through IL‐4, induce IgE production by B cells through IL‐4 and IL‐13, participate in eosinophil activation and IgE synthesis, and promote the recruitment of innate cells to the site of inflammation.^20^ In addition, Ezrin can regulate mast cell degranulation^21^ and T cell morphology, and dephosphorylation of Ezrin triggers the rapid collapse of T lymphocyte microvilli, resulting in the arrest of circulating T lymphocytes.^22^ It has been found that recombinant wild‐type Ezrin could significantly reduce the sensitivity of mitochondrial permeability transition pores to calcium.^23^ Ezrin can co‐locate with CD44, which is necessary for mitochondrial function, and overexpression of Ezrin can increase the potential of the inner mitochondrial membrane and protect the structure of the damaged mitochondria.^24^ Unfortunately, limited research has been conducted on the role of Ezrin in inflammatory diseases, including AD.

In recent years, increasing attention has been paid to alternative therapeutic drugs for AD, especially natural bioactive compounds derived from plant extracts. Celastrol is a natural triterpenoid compound extracted from the root bark of the medicinal plant *Tripterygium wilfordii*. It has various pharmacological activities, such as anti‐inflammatory, anti‐obesity,^25^ antioxidant,^26^ and anticancer (including cancer metastasis) effects.^27^ It has been widely used in China and other Asian countries for the treatment of various diseases, including chronic inflammation and immune diseases.^28^ Celastrol has been reported to inhibit ROCK2‐mediated phosphorylation of Ezrin at Thr567 in hepatocellular carcinoma^29^ and can inhibit mitochondrial division by promoting the phosphorylation of Drp1 at Ser637.^27^ In addition, celastrol can improve mitochondrial function by activating the PI3K‐Akt signalling pathway in C2C12 myotubes and significantly reduce cisplatin‐induced mitochondrial ROS (mtROS) accumulation.^30^ However, the impact of celastrol on mitochondrial dynamics is still unclear. Celastrol has been shown to improve erythema and scales on the dorsal skin of psoriatic mice, increase HaCaT cell activity, reduce cellular apoptosis and inhibit the release of inflammatory factors such as IL‐6, IL‐22, IL‐23 and IL‐17.^31^ It is still unclear whether celastrol has similar therapeutic effects on AD.

Herein, we aimed to investigate the therapeutic effects of celastrol on AD and the possible mechanisms involving Ezrin and mitochondrial fission and fusion. Our results herein indicate that celastrol could alleviate AD by regulating Ezrin‐mediated mitochondrial fission and fusion. Therefore, celastrol is expected to become a natural drug for the treatment of AD.

## 2 MATERIALS AND METHODS

### 2.1 Animals and cells

Totally 30 female Balb/c mice (6–8 weeks old, SPF grade) were purchased from the Experimental Animal Center of Yanbian University (Licence no. SCXK (Ji) 2017‐0003). Animals were kept under standard conditions. Animal experimental procedures complied with the ARRIVE guidelines and were approved by the Ethics Committee of the Jilin Provincial Department of Science and Technology (approval no. SYXK (Ji) 2020 009).

### 2.2 Animal model establishment and grouping

The AD model was induced with 2,4‐dinitrochlorobenzene (DNCB).^32^ Briefly, after acclimation for 1 week, the Balb/c mice were randomly divided into the control, model, celastrol (25, 50 and 75 μg) and Mdivi‐1 (a mitochondrial fission inhibitor; 25 mg/kg) groups (*n* = 5), respectively. The 1% DNCB (237329‐10G; Alorich Merck, NJ, USA) was dissolved in a mixture of acetone (GB686‐89; Tianjin Quartz Clock Factory Bazhou Chemical Factory, Bazhou, China) and olive oil (69,018,028; China National Pharmaceutical Group Chemical Reagent Co., Ltd., Xi'an, China) (3:1 v/v). For the model establishment, 200 μL of 1% DNCB was applied locally on the dorsal skin and 20 μL on the ear, three times a week for 3 weeks, sensitising for 1 week and then resting for 1 week. Starting from week 3, 0.5% DNCB dissolved in acetone and olive oil was used for the AD challenge in each group (three times a week for 3 weeks). In the celastrol groups, 25 μg, 50 μg and 75 μg celastrol (C2218431; Aladdin, Beijing, China) were directly added to the dissolved DNCB. The Mdivi‐1 group was intraperitoneally injected with Mdivi‐1 (25 mg/kg/d; C2218431; Aladdin; Beijing, China), which was dissolved in a mixture of DMSO and saline (1:9 v/v). The other groups were intraperitoneally injected with the same volume of DMSO and saline mixture.

### 2.3 Sample collection

On the second day after model establishment, the mice were anaesthetized with ether, and the mouse spleen and lymph nodes, as well as the ear and dorsal skin tissues, were removed and collected. The thickness of the ear and dorsal skin tissues was measured using a thickness gauge (Weihai Xinwei Measuring Tool Co., Ltd., Weihai, China). The spleen and lymph nodes were weighed. The spleen index and lymph node index were calculated using the following formula: Organ index = Organ weight/Body weight × 100%.

### 2.4 Haematoxylin and eosin (HE) and toluidine blue staining

The tissue was fixed with 4% paraformaldehyde (P1110‐100 mL; Solarbio, Beijing, China) for 1 week, which was then embedded in paraffin and cut into 4‐μm sections. The tissue sections were stained with HE (G1120; Solarbio) and toluidine blue (G3670; Solarbio), respectively. Then the images were analysed with the Slide scanning system (SQS‐40R; Shengqiang Technology, Shenzhen, Guangdong, China). The number of mast cells was counted in three randomly selected fields under the same magnification.

### 2.5 Immunohistochemical staining

The tissue sections were incubated with 10% goat serum for 30 min. Then the samples were incubated with the anti‐Drp (ab184247; Abcam, Cambridge, UK), anti‐P‐Drp1 (3455S; Cell Signalling Technology, Boston, UK), anti‐Ezrin (ab4069; Abcam) and anti‐P‐Ezrin (ab47293; Abcam) primary antibodies, respectively, at 4°C for 24 h. Subsequently, all samples were incubated with the mouse/rabbit‐enhanced polymer detection system (PV‐9000; Zhongshan Golden Bridge Biotechnology Co., Ltd., Beijing, China). The sections were observed with the slide scanning system (# SQS‐40R; Shengqiang Technology).

### 2.6 Flow cytometry detection

Cells were extracted from mouse spleen and lymph node tissues, and the live cells were selected with the eBioscience™ Fixable Viability Dye eFluor™ 780 (65‐0865‐14; Invitrogen, Carlsbad, CA, USA). Then the cells were incubated with the anti‐CD4 (Mouse) mAb‐FITC (D341‐4; MBL, Ina‐shi Nagano‐kens, Japan), anti‐mouse IL‐4 APC (20‐7041‐0025; TONBO bioscience, San Diego, CA, USA) and APC anti‐mouse tumour necrosis factor‐α (TNF‐α) (506,308; Biogene, San Diego, CA, USA) antibodies, or with anti‐Mo IL‐13 eBioscience™ APC‐eFluor 780 (47‐7133‐80; Invitrogen), respectively. Finally, cells were analysed on the Cytoflex flow cytometer (A00‐1‐1102; Beckman Coulter), and the data were analysed using the Cytoexpert 2.4 software (Beckman Coulter).

### 2.7 Cell line

The HaCaT cells were purchased from Fenghui Biotechnology Co., Ltd. (CL0114; Changsha, China). The cell line was cultured in a modified DMEM medium (C3110‐0500 L; VivaCell, Shanghai, China) containing 10% foetal bovine serum (040011ACS‐50 mL; Biological Industries, Kibbutz Beit Haemek, Israel), supplemented with 10,000 U/L and 10 mg/mL Pen‐Strept Solution.

### 2.8 Cell treatment

HaCat cells were stimulated with 10 ng/mL TNF‐α (300‐01A‐10UG; Peprotech, Princeton, USA) for 24 h. Celastrol (0.25 μM, 0.5 μM and 1 μM; 34,157‐83‐0; Solarbio, Beijing, China) and Mdivi‐1 (2.5 μM, 5 μM, 7.5 μM and 10 μM; HY‐15886, MedChemExpress, Shanghai, China) were added to the cell culture medium 2 h before TNF‐α induction.

### 2.9 Co‐immunoprecipitation (COIP)

The HaCat cells were lysed, and the lysate was incubated with Drp1‐immobilised resin. After overnight incubation at 4°C, the samples were washed, collected and subjected to Western blot analysis.

### 2.10 MTT assay

The HaCaT cells were seeded onto the 96‐well plate at a density of 1 × 10^4^ cells/well and treated with 0, 0.1, 0.25, 0.5, 1, 2.5 and 5 μM celastrol, respectively, at 37°C for 24 h. Followingly, the cells were treated with 7 μL MTT (5 mg/mL, dissolved in DMSO; M8180‐250 mg; Solarbio) for 3 h. The absorbance at 450 nm was measured using a microplate reader.

### 2.11 Cell transfection

HaCat cells were transfected with Ezrin‐specific siRNA (stB0002569; RIBOBIO, Guangzhou, China) using Lipofectamine 3000 (L3000‐015; Invitrogen). After 48 h of transfection, the cells were treated and stimulated with TNF‐α for 24 h.

### 2.12 ELISA

The mouse skin tissues were ground into homogenate, which was centrifuged at ×3500 *g* at 4°C for 10 min. IgE (EK275, MULTI SCIENCES, Hangzhou, China) in mouse serum, levels of IL‐5, IL‐17, IFN‐γ (900‐K406, 900‐K392, 900‐T98; PEPROTECH, Princeton, USA), IL‐31 (ab243681; Abcam, Discovery Drive Cambridge Biomedical Campus Cambridge CB2 0AX, United Kingdom), IL‐33, Thymic Stromal Lymphopoietin (TSLP), eosinophil chemotactic protein (ie CCL11), IL‐1β and IL‐23 (EK233, EK265, EK2130, EK201B, EK223; MULTI SCIENCES, Hangzhou, China), thymic and activating regulatory chemokine (TARC, ie CCL17) (ml057939; Mlbio, Shanghai, China) in the mouse skin tissue homogenate, as well as the levels of IL‐4, IL‐5, IL‐13, TSLP and IL‐1β (900‐T14, 900‐K15, 900‐K334, 900‐T14, 900‐K95; PEPROTECH, Princeton, USA) in the culture supernatants of HaCat cells, were measured with corresponding ELISA kits.

### 2.13 Western blot analysis

Mouse skin tissues and HaCat cells were lysed with RIPA (R0020; Solarbio) and PMSF (P0100‐1 mL; Solarbio). Protein concentrations were qualified with the BCA detection kit (# PC0020; Solarbio). Totally 30 μg protein was separated by 10% SDS‐PAGE and then electrophoretically transferred onto a PVDF membrane (1SEQ00010 + IPVH00010; Millipore, Ireland). After blocking, the membrane was incubated with primary antibodies against Ezrin (ab4069; Abcam), P‐Ezrin (ab47293; Abcam), Drp1 (611,738; Beckon Dickinson, New Jersey, USA), Drp (ab184247; Abcam), P‐Drp1 (3455S; Cell Signalling Technology), Fis1 (ab229969; Abcam), Mfn1 (# DF7543; Affinity, Burnet Ave, USA) and Mfn2 (11925S; Cell Signalling Technology), respectively, at 4°C overnight. Then the membrane was incubated with the corresponding secondary antibodies. Colour development was performed with ECL. Protein expression levels were analysed using the Image J software. β‐actin was used as an internal reference.

### 2.14 Immunofluorescence staining

Mouse skin tissue sections were repaired in citrate buffer solution at pH 6.0, which were then incubated with antibodies against Ezrin (ab4069; Abcam), P‐Ezrin (ab47293; Abcam), Drp1 (611,738; Beckon Dickinson) and translocase of outer mitochondrial membrane 20 (TOMM20) (ab186735; Abcam). HaCat cells were incubated with antibodies against Ezrin (ab4069; Abcam), P‐Ezrin (ab47293; Abcam), Drp (ab184247; Abcam), P‐Drp1 (3455S, Cell Signalling Technology) and TOMM20 (ab186735; Abcam), respectively. Then the sections and cells were incubated with Alexa Fluor™ 488 goat anti‐mouse IgG (H + L) (A1101; Invitrogen) CY3‐coupling (S001; Affinity), Goat Anti‐Rabbit IgG H&L (HRP) preadsorbed (ab97035; Abcam) and anti‐rabbit IgG Fab2 Alexa Fluor (R) 488 Molecular Probes (4412S; Cell Signalling Technology). All samples were imaged with Cytation 5.

### 2.15 Mitochondrial morphology determination

Cells were incubated with MitoTracker™ Red CMXRos (M7512; Invitrogen) at 37°C for 30 min, followed by Cytonation 5 imaging or cell fixation, permeabilization and incubation overnight with anti‐Drp1 antibody (ab184247; Abcam) at 4°C. Then, the samples were incubated with anti‐rabbit IgG Fab2 Alexa Fluor (R) 488 Molecular Probes (4412S; Cell Signalling Technology) for 2 h. Imaging was performed with Cytation 5.

### 2.16 Detection of mtROS

Cells were incubated with MitoSox™ Red mitochondrial superoxide indicator (M36008; Invitrogen) at 37°C for 30 min, followed by Cytology 5 imaging or cell fixation, permeabilization and overnight incubation with anti‐TOMM20 antibody (ab186735; Abcam) at 4°C. Anti‐rabbit IgG Fab2 Alexa Fluor (R) 488 Molecular Probes (4412S; Cell Signalling Technology) were used for incubation for 2 h. Finally, imaging was performed with Cytation 5.

### 2.17 Mitochondrial membrane potential (MMP) determination

The MMP was determined with the mitochondrial membrane potential assay kit with JC‐1 (5 μM; C2006; Beyotime, Shanghai, China). Cytation 5 was used for imaging.

### 2.18 ROS detection

Cells were incubated with ROS‐specific fluorescent probe DCFH‐DA (10 μM; S0033S; Beyotime) at 37°C for 30 min. Then cells were observed with Cytation 5.

### 2.19 Statistical analysis

Statistical analysis was performed using Prism7.0 software. Data are presented as mean ± standard deviation. Comparisons between groups were performed by *t*‐test and one‐way analysis of variance. *p* Values <0.05 were considered statistically significant.

## 3 RESULTS

### 3.1 Mdivi‐1 reduces TNF‐α‐induced inflammatory response in HaCaT cells

To verify the regulatory effect of Drp1 in AD, the HaCat cells were intervened with Mdivi‐1. Our results showed that Mdivi‐1 (2.5, 5, 7.5 and 10 μM) selectively inhibited the binding of Drp‐1 to mitochondria. MitoTracker Red staining showed that, compared with the Model group, Mdivi‐1 inhibited the TNF‐α‐induced transfer of Drp1 to the mitochondrial membrane (Figure S1A). Moreover, Western blot analysis revealed that Mdivi‐1 decreased the phosphorylation of the mitochondrial fission protein Drp1 and the expression of Fis1, while it increased the expression of the mitochondrial fusion proteins Mfn1 and Mfn2 (Figure S1B). There was no significant difference in Opa1 protein expression. Additionally, as revealed by ELISA and MitoSOX Red staining, Mdivi‐1 significantly inhibited the levels of IL‐4, IL‐5, IL‐13 and TSLP in the supernatant of HaCat cells (Figure S1C) and decreased mtROS production (Figure S1D). The 10 μM Mdivi‐1 had the best effect. These results suggest that Mdivi‐1 reduces TNF‐α‐induced inflammatory response in HaCaT cells.

### 3.2 Ezrin regulates mitochondrial fission and fusion and mtROS production in HaCaT cells

**FIGURE 1:**
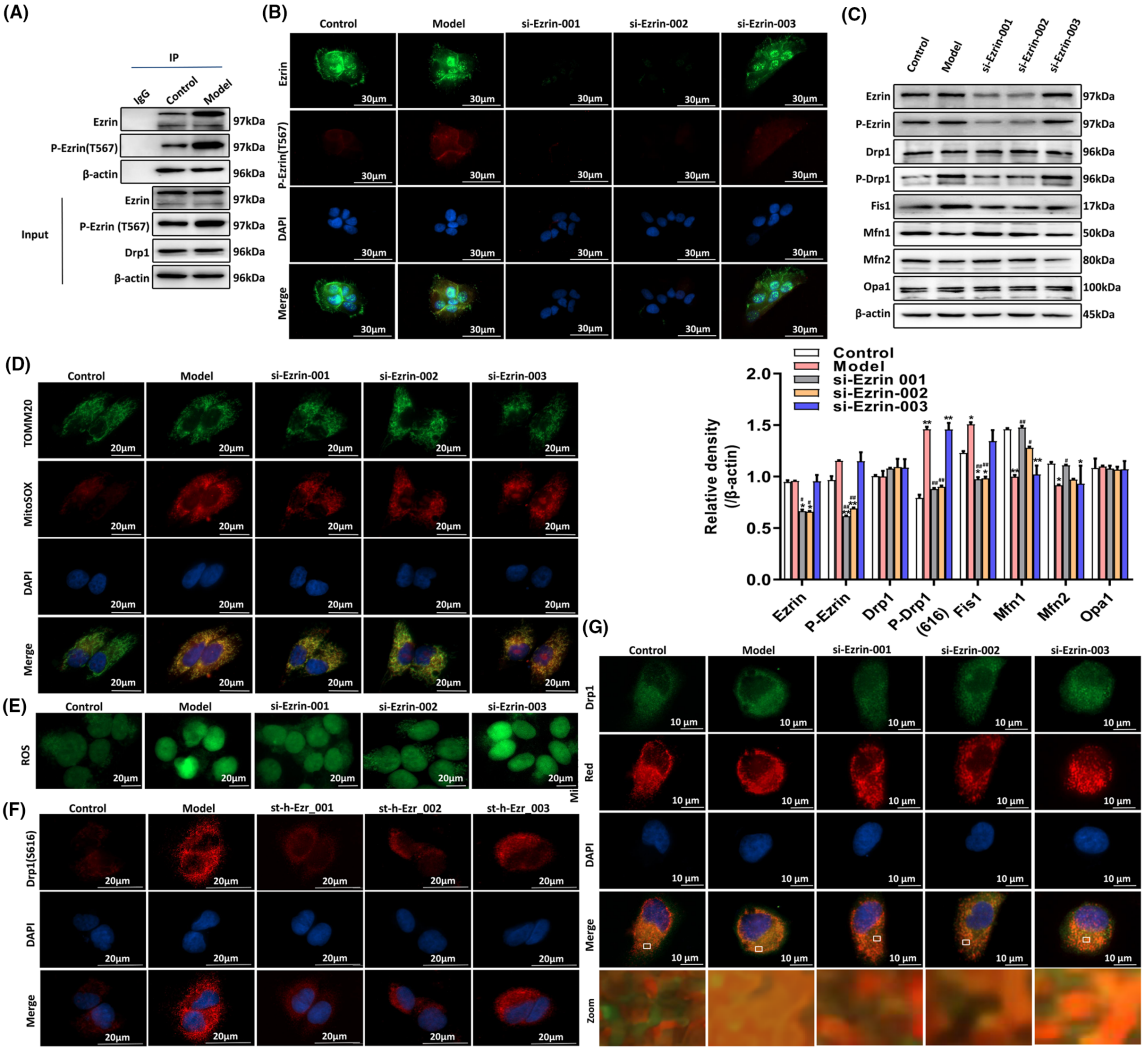
Ezrin regulates mitochondrial fission and fusion and mtROS production in cells. Three Ezrin‐specific siRNAs (the si‐Ezr‐001, si‐Ezr‐002 and si‐Ezr‐003) were transfected into HaCat cells for 48 h. (A) COIP was used to observe the specific binding of Ezrin, P‐Ezrin (T567) and Drp1. (B) Immunofluorescence staining was used to observe the localization and expression of Ezrin and P‐Ezrin (T567) in HaCat cells. (C) Western blot analysis was used to measure the expression of Ezrin, P‐Ezrin (T567), Drp1, P‐Drp1, Fis1, Mfn1, Mfn2 and Opa1. (D) Observation of mitochondrial morphology and mtROS by immunofluorescence staining of TOMM20 and MitoSOX. (E) ROS generation was observed after DCFH‐DA staining. (F) Cellular immunofluorescence staining was used to observe the localization and expression of P‐Drp1. (G) Mitotracker Red and Drp1 fluorescence staining were used to observe the translocation of Drp1 to mitochondria. *p < 0.05 and **p < 0.01, compared to the Control group; and #*p* < 0.05 and ##*p* < 0.01, compared with the Model group.

To investigate the interaction between Drp1 and Ezrin, the COIP was performed. As shown in Figure 1A, Ezrin and P‐Ezrin (T567) could bind to Drp1. To knock down Ezrin expression, three Ezrin‐specific siRNAs were constructed, that is, the si‐Ezrin‐001, si‐Ezrin‐002 and si‐Ezrin‐003. Immunofluorescence and Western blot analysis showed that the si‐Ezrin‐001 and the si‐Ezrin‐002 successfully knocked down the expression of Ezrin (Figure 1B,C). After knocking down Ezrin, the expression of P‐Drp1 (S616) and Fis1 was significantly decreased, while the expression levels of Mfn1 and Mfn2 were significantly increased (Figure 1B,C,F). MitoSox (Figure 1D), DCFH‐DA (Figure 1E), and MitoTracker staining (Figure 1G) showed a decrease in mtROS and ROS production after knockout of Ezrin (Figure 1D,E) and restored TNF‐α‐induced mitochondrial disruption, resulting in reduced expression of mitochondrial fission protein Drp1 (Figure 1F), which is similar to the role of Mdivi‐1 in inhibiting Drp1. These results suggest that Ezrin may regulate mitochondrial fission and fusion and mtROS production by affecting the level of mitochondrial fission and fusion proteins.

### 3.3 Celastrol inhibits Ezrin activation in HaCaT cells

**FIGURE 2:**
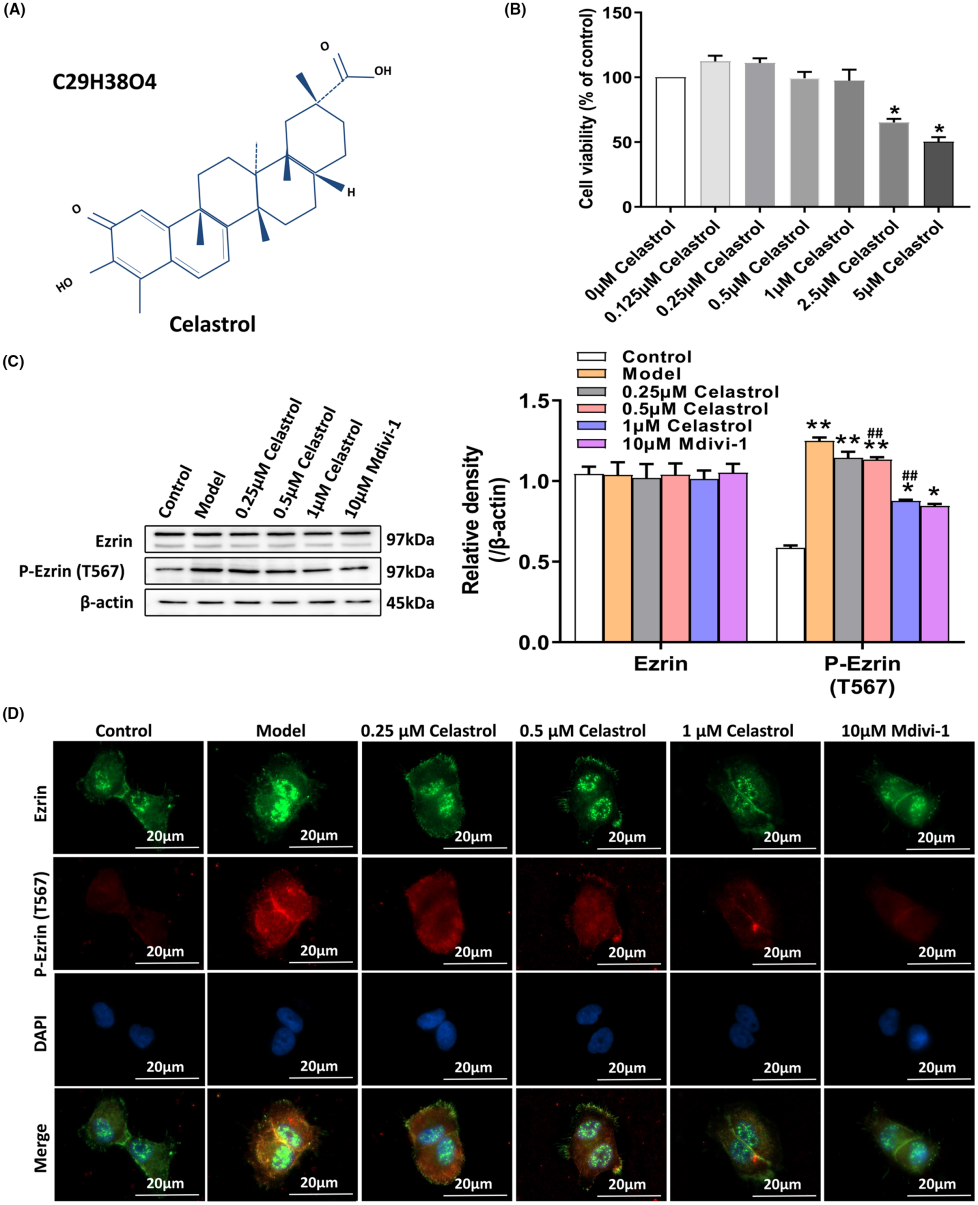
Celastrol inhibits the activation of Ezrin in HaCaT cells. (A) The molecular formula of celastrol. (B) Different concentrations of celastrol (0, 0.125, 0.5, 1, 2.5 and 5 μM) were used to pre‐treat HaCaT cells, and the cell viability was measured with the MTT assay. (C) Western blot analysis was used to measure the expression of Ezrin and P‐Ezrin (T567). (D) Cellular immunofluorescence staining of Ezrin and P‐Ezrin (T567). *p < 0.05 and **p < 0.01, compared to the Control group; and #*p* < 0.05 and ##*p* < 0.01, compared with the Model group.

The molecular formula of celastrol was C_29_H_38_O_4_ (Figure 2A). The MTT assay was used to determine the effect of celastrol on the proliferation of HaCaT cells. As shown in Figure 2B, compared with the control group (0 μM), 2.5 μM and 5 μM celastrol resulted in significantly lower cell viability. The cell viability was not significantly changed by celastrol at other concentrations. Therefore, the concentrations of 0.25, 0.5 and 1 μM were used for subsequent experiments.

To determine the regulatory effect of celastrol on Ezrin in HaCaT cells, we performed western blot analysis and immunofluorescence staining. The expression levels of total Ezrin and P‐Ezrin (T567) were detected. As shown in Figure 2C,D, after the celastrol treatment, compared with the Model group, the expression level of P‐Ezrin (T567) was sharply decreased in a dose‐dependent manner, similar to the Mdivi‐1 treatment group. The expression of P‐Ezrin (T567) on the membrane of HaCaT cells was reduced after the celastrol treatment. The above results indicate that celastrol inhibits the activation of Ezrin in the AD cell model.

### 3.4 Celastrol attenuates phosphorylation of Drp1 at Ser616 in HaCaT cells

**FIGURE 3:**
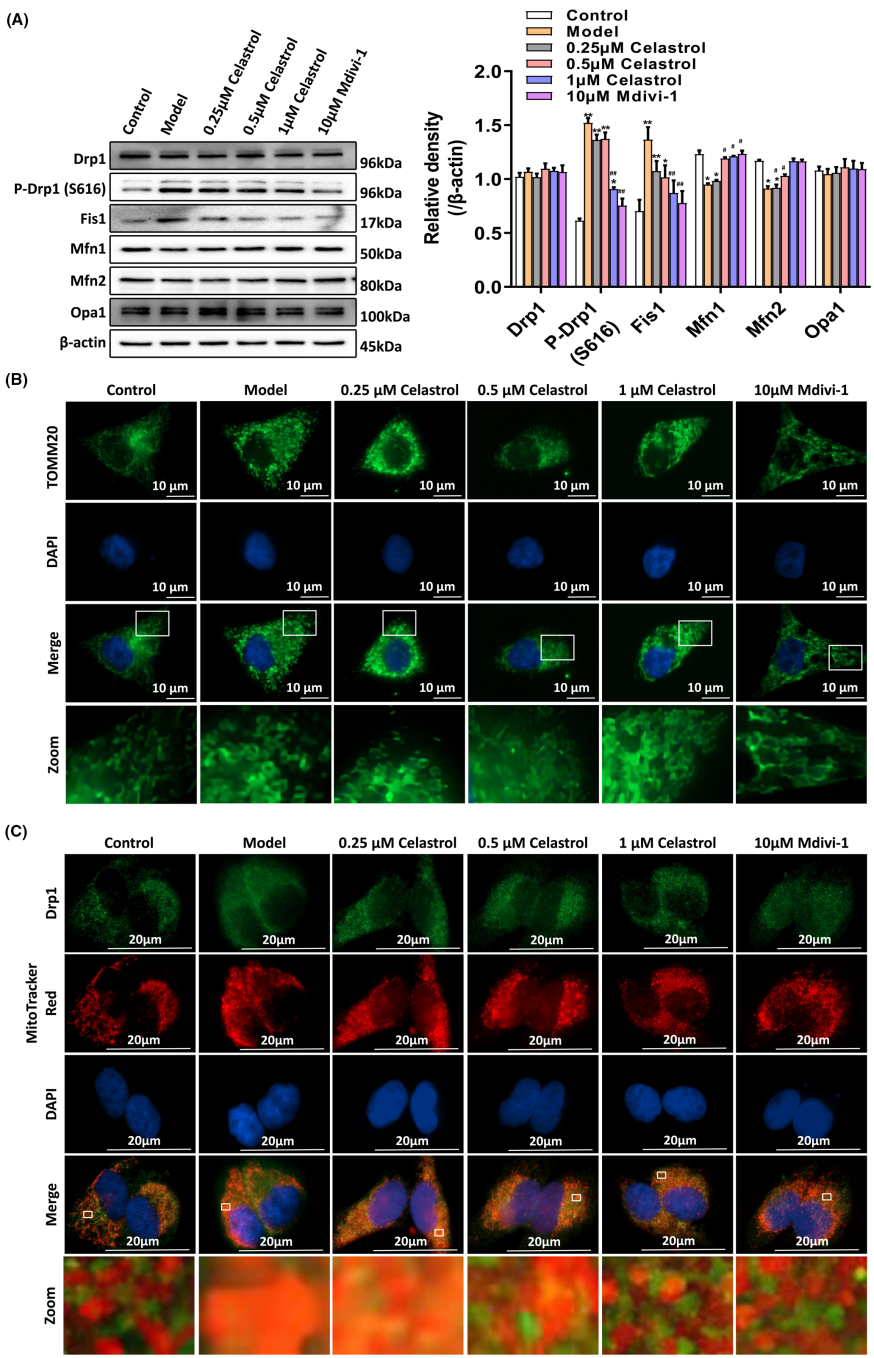
Celastrol attenuates phosphorylation of Drp1 at the Ser616 in HaCaT cells. (A) Western blot analysis was used to measure the expression of Drp1, P‐Drp1, Fis1, Mfn1, Mfn2 and Opa1. (B) Cellular immunofluorescence staining of TOMM20 was used to observe the morphological changes of mitochondria. (C) Mitotracker Red and Drp1 fluorescence staining were used to observe the translocation of Drp1 to mitochondria. *p < 0.05 and **p < 0.01, compared to the Control group; and #*p* < 0.05 and ##*p* < 0.01, compared with the Model group.

To investigate the regulation of Drp1 by celastrol in HaCaT cells, we performed the Western blot analysis. Moreover, the mitochondrial morphological changes were observed by immunofluorescence. As shown in Figure 3A,B, the celastrol pretreatment reduced the TNF‐α‐stimulated Drp1 phosphorylation at Ser616, increased the expression of Mfn1 and Mfn2 and restored the mitochondrial network structure. MitoTracker Red and Drp1 staining showed a decrease in mitochondrial Drp1 expression after the celastrol pretreatment, and Drp1 returned to the cytoplasm (Figure 3C). Celastrol displayed similar effects to the Mdivi‐1 positive control group. We believe that celastrol may primarily regulate mitochondrial dynamics by inhibiting the phosphorylation of Drp1 at Ser616.

### 3.5 Celastrol reduces mtROS production and restores MMP in HaCaT cells

The effects of celastrol on mtROS and ROS production in HaCaT cells were detected with the MitoSOX Red staining and DCFH‐DA staining. We found that, consistent with the Mdivi‐1 treatment, the celastrol treatment induced an obvious decrease in mtROS and ROS levels (Figure S2A,B). JC‐1 staining showed that TNF‐α‐treated cells showed a lack of MMP, in which the accumulation of JC‐1 in mitochondria was inhibited and the formation of J‐aggregates was reduced, resulting in an evident shift from red to green fluorescence (Figure S2C). After the celastrol or Mdivi‐1 pre‐treatment, the JC‐1 probes formed J‐aggregates in the mitochondria, generating bright red fluorescence and thus restoring MMP.

### 3.6 Celastrol inhibits Ezrin activation in the skin tissues of AD mice

**FIGURE 4:**
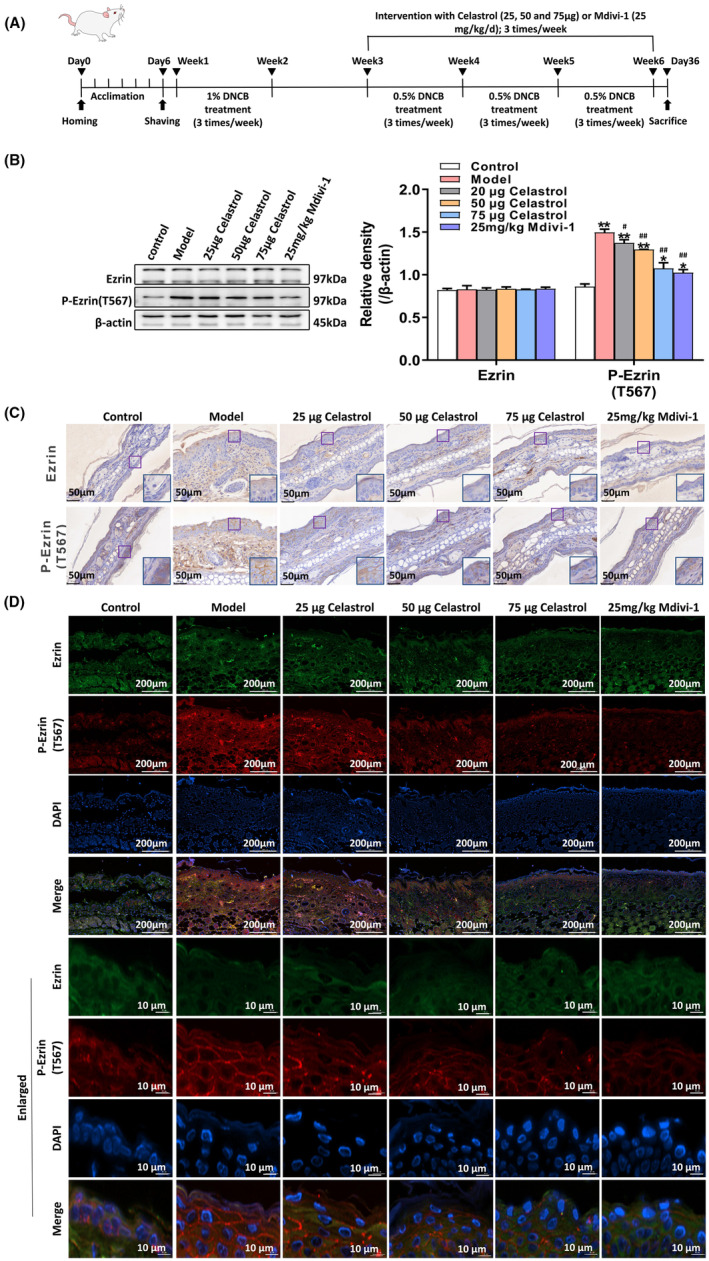
Celastrol inhibits Ezrin activation in the skin tissues of AD mice. (A) Experimental procedure for AD model establishment. (B) Western blot analysis was used to measure the expression of Ezrin and P‐Ezrin (T567). (C) Immunochemical staining was used to observe the localization and expression of Ezrin and P‐Ezrin (T567) in the ear skin tissue of the AD model. (D) Observation of the localization and expression changes of Ezrin and P‐Ezrin (T567) in the AD model using tissue fluorescence staining. *p < 0.05 and **p < 0.01, compared to the Control group; and #*p* < 0.05 and ##*p* < 0.01, compared with the Model group.

To investigate whether celastrol has the same regulatory effect on Ezrin in the skin of AD, the Balb/c mouse AD model was established (Figure 4A), and the Western blot analysis and immunohistochemical staining were used to detect the expression and activation of Ezrin protein. As shown in Figure 4B,C, there was a dose‐dependent decrease in Ezrin phosphorylation levels at Thr 567 in the skin tissue of Balb/c mice in the celastrol groups than in the model group, with the concentration of 75 μg celastrol exerting the best effects. The effect of celastrol on Ezrin phosphorylation was similar to that of Mdivi‐1. Immunofluorescence staining was used to detect the localization of total Ezrin and P‐Ezrin (T567) in mouse skin. As shown in Figure 4D, inactive Ezrin was distributed in the cytoplasm of the skin epidermal cells, while activated P‐Ezrin (T567) was present on the epidermal cell membrane. Thus, celastrol could inhibit Ezrin activation in the skin epidermal cells of AD mice.

### 3.7 Celastrol regulates mitochondrial fission and fusion proteins in AD mice

**FIGURE 5:**
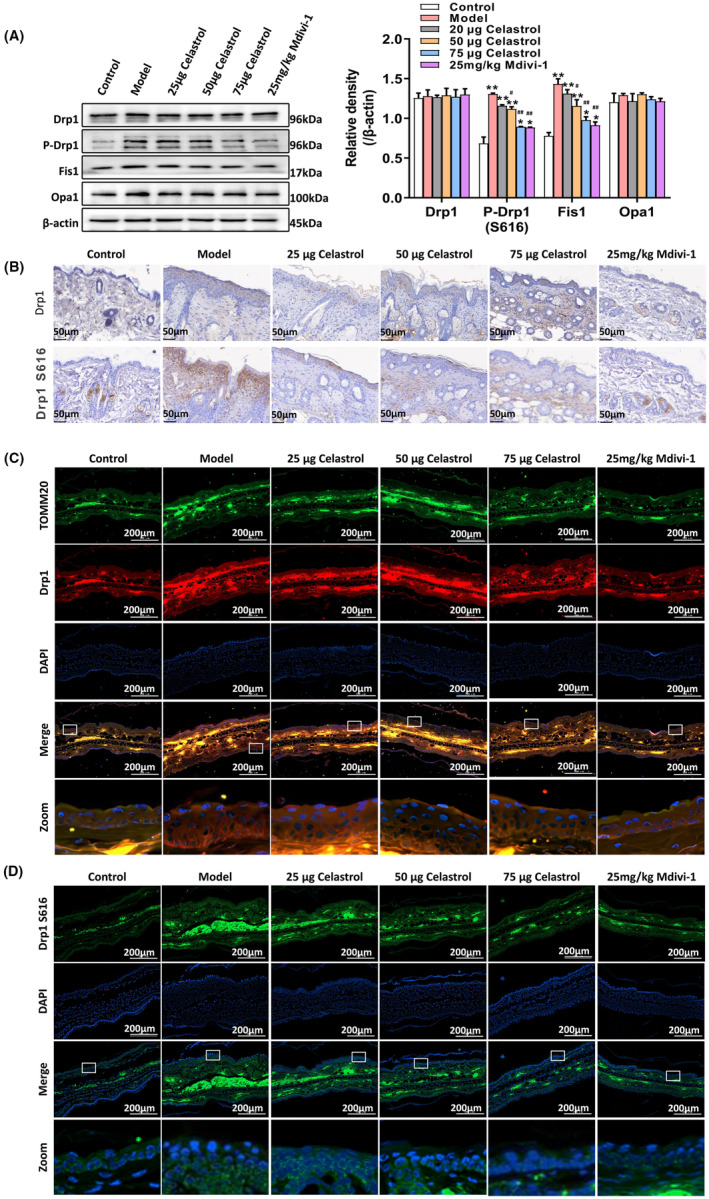
Celastrol regulates the expression of mitochondrial fission and fusion proteins in AD mice. (A) Western blot analysis was performed to detect the expression of Drp1, P‐Drp1, Fis1 and Opa1. (B) Immunochemical staining was used to observe the localization and expression changes of Drp1 and P‐Drp1 in the dorsal skin tissue of the AD model. (C) Observation of the localization and expression changes of TOMM20 and Drp1 in ear skin tissue using tissue fluorescence staining. (D) Observation of the localization and expression changes of P‐Drp1 (S616) in ear skin tissue using tissue fluorescence staining. *p < 0.05 and **p < 0.01, compared to the Control group; and #*p* < 0.05 and ##*p* < 0.01, compared with the Model group.

To investigate the regulation of mitochondrial dynamics by celastrol in AD, we performed Western blot analysis, immunohistochemical staining and immunofluorescence staining. As shown in Figure 5A,B, compared with the model group, the celastrol treatment and the Mdiv‐1 treatment alleviated the imbalance of P‐Drp1, Fis1, Mfn1 and Mfn2 expression and reduced the expression of P‐Drp1 (S616) in AD mice. Co‐localization of Drp1 and mitochondrial outer membrane marker TOMM20 was observed in the mouse ear skin tissue of the model group (Figure 5C). In the celastrol treatment group, most Drp1 is located in the cytoplasm (Figure 5C). This was further confirmed by immunofluorescence staining of P‐Drp1 (S616) (Figure 5D). These results suggest that celastrol may have a regulatory effect on mitochondrial fission and fusion, possibly by inhibiting the activation of Ezrin.

### 3.8 Local application of celastrol improves DNCB‐induced skin thickening and spleen and lymph node weight increase in AD mice

**FIGURE 6:**
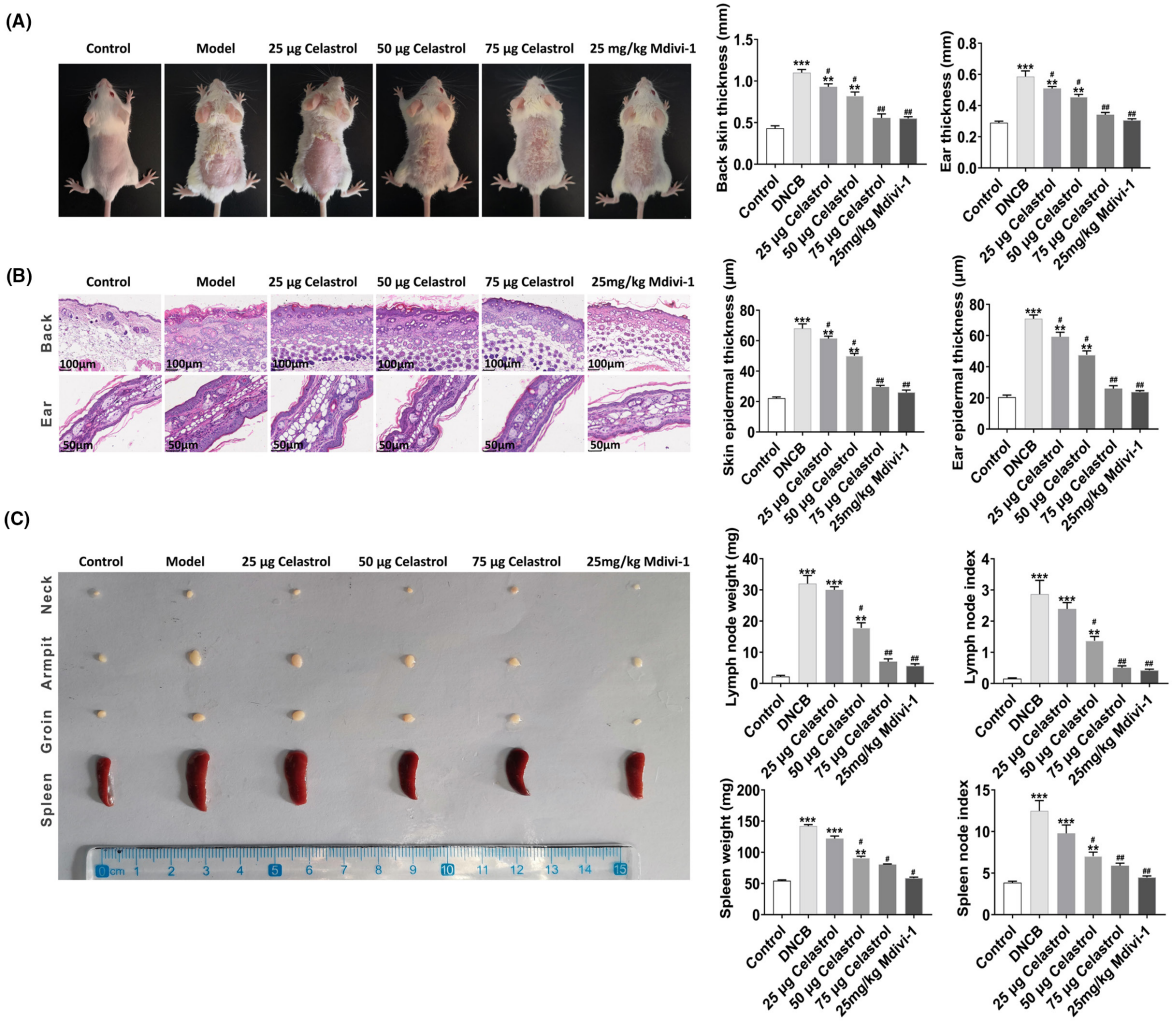
Local application of Celastrol improves the skin thickening and spleen and lymph node weight increase induced by DNCB in Balb/c mice. (A) On the last day of the model establishment, the skin changes were observed, and the thickness of the dorsal and ear skin tissues was shown on the right panel. (B) HE staining was used to observe the pathological changes. The epidermal thickness was shown on the right panel. (C) The gross morphology (left panel) and the organ index (right panel) of the spleen and lymph nodes. *p < 0.05 and **p < 0.01, compared to the Control group; and #*p* < 0.05 and ##*p* < 0.01, compared with the Model group.

To evaluate the therapeutic effect of celastrol on AD, we first evaluated the gross morphology and the pathological changes of mouse skin. The results showed that the celastrol or Mdivi‐1 treatment reduced AD‐like lesions, such as bleeding, oedema, epidermal detachment and scales in mice, reduced the ear and dorsal skin thickness (*p* < 0.001) and decreased inflammatory cell infiltration (Figure 6A,B). Furthermore, we assessed the spleen and lymph nodes of AD mice. Our results showed that the weights and organ indexes of the spleen and lymph nodes in the celastrol or Mdivi‐1 treatment group were significantly decreased than in the model group (Figure 6C). The best effects of celastrol were achieved at 50 and 75 μg. These data imply that local application of celastrol restores skin thickness and weakens the infiltration of inflammatory cells, exerting anti‐AD effects in mice.

### 3.9 Celastrol has therapeutic effects on AD‐related inflammatory cytokines and degranulation of mast cells in AD mice

**FIGURE 7:**
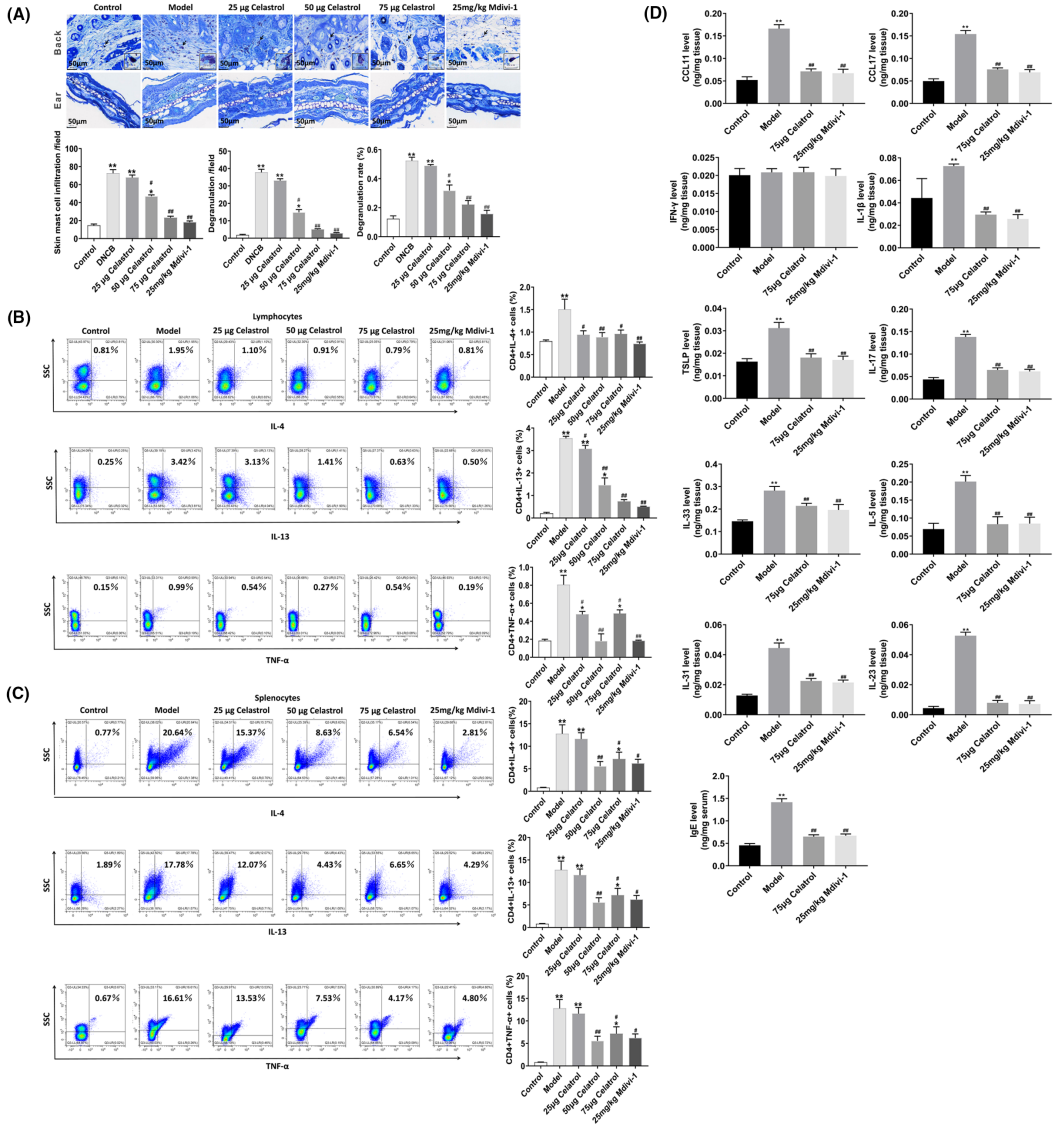
Celastrol has a therapeutic effect on AD‐like symptoms and degranulation of mast cells in Balb/c mice. (A) Toluidine blue staining was used to observe the infiltration and degranulation of mast cells in the dorsal skin and ear skin of mice. The mast cell infiltration, degranulation number and degranulation rate of mast cells were shown on the lower panel. (B) Detection of Th2 cytokines IL‐4, IL‐13 and TNF‐α in mouse lymph nodes by flow cytometry. (C) Detection of Th2 cytokines IL‐4, IL‐13 and TNF‐α in mouse spleen cells by flow cytometry. (D) ELISA detection of chemokines CCL11, CCL17 and cytokine IFN‐γ in mouse skin homogenate, as well as the expression of IL‐1β, TSLP, IL‐17, IL‐33, IL‐5, IL‐31, IL‐23 and IgE. *p < 0.05 and **p < 0.01, compared to the Control group; and #*p* < 0.05 and ##*p* < 0.01, compared with the Model group.

The toluidine blue staining was used to observe the mast cells in the skin of AD mice. Our results showed that, after the celastrol or Mdivi‐1 treatment, the infiltration of mast cells was reduced and the degranulation was significantly improved (Figure 7A). Flow cytometry and ELISA were used to detect the effects of celastrol on the production of AD‐related cytokines in the spleen, lymph nodes and skin homogenate of AD mice. The IL‐4, IL‐13 and TNF‐α levels were significantly reduced in the spleen and lymph nodes of AD mice after the celastrol or Mdivi‐1 treatment (Figure 7B,C). Moreover, the chemokines of CCL11 and CCL17, as well as the cytokines IL‐1β, TSLP, IL‐17, IL‐33, IL‐5, IL‐31, IL‐23 and IgE, were significantly decreased. However, no significant changes were observed in the Th1‐mediated cytokine IFN‐γ between the control and model groups. Therefore, celastrol could inhibit the degranulation of mast cells in AD mice and reduce the expression of AD‐related inflammatory cytokines.

## 4 DISCUSSION

Multiple genes are dysregulated in AD, especially Th2‐related genes (such as IL‐4, IL‐10 and IL‐13).^33^ Mdivi‐1 selectively inhibits the mitochondrial fission protein Drp1 by blocking the self‐assembly of Drp1 and exerts therapeutic effects in a variety of diseases.^10^ Li et al.^34^ found that in AD mice, the Mdivi‐1 treatment could improve the AD‐like symptoms of mice, as manifested by the reduction of serum IgE levels, the thickening of the epidermis, the infiltration of mast cells, and the reduced production of IL‐4, IL‐5 and IL‐13. Our results found that Mdivi‐1 inhibited the translocation of Drp1 to mitochondria, the production of mtROS and the TNF‐α‐induced release of IL‐4, IL‐5, IL‐13, TSLP and IL‐1β in HaCat cells. Based on these findings, we believe that inhibiting mitochondrial fission and fusion may become a new strategy for the treatment of AD.

Ding et al.^35^ found that Ezrin promoted the generation of inflammatory factors TNF‐α, IL‐1β and HMGB1. Zhao et al.^36^ showed that the Ezrin phosphorylation limited the production of IL‐10 in B cells. Shan et al.^37^ believed that the cytoskeleton interaction mediated the mitochondrial dynamics and localization. In this study, we found that Ezrin could bind specifically to Drp1. We also found that interfering with the expression of Ezrin inhibited the translocation of Drp1 from the cytoplasm to mitochondria, inhibited the expression of mitochondrial fission protein and promoted the expression of the mitochondrial fusion protein, thereby regulating mitochondrial dynamics and restoring the mitochondrial reticular formation, cellular ROS and mt ROS generation. This demonstrates that Ezrin can regulate the fission and fusion of mitochondria.

Here, we detected the expression of Ezrin and mitochondrial fission and fusion protein in the AD cell model and found that celastrol inhibited the activation of Ezrin, the phosphorylation of mitochondrial Drp1 at the Ser616 and the expression of Fis1, while up‐regulating the Mfn1 and Mfn2. Celastrol not only regulated the mitochondrial dynamics and maintained mitochondrial network structure but also inhibited the production of mtROS and ROS and restored MMP. This is consistent with the results following the inhibition of mitochondrial fission by Mdivi‐1 and Ezrin knockdown. Therefore, celastrol may exert its effects on mitochondria by regulating Ezrin activation.

**FIGURE 8:**
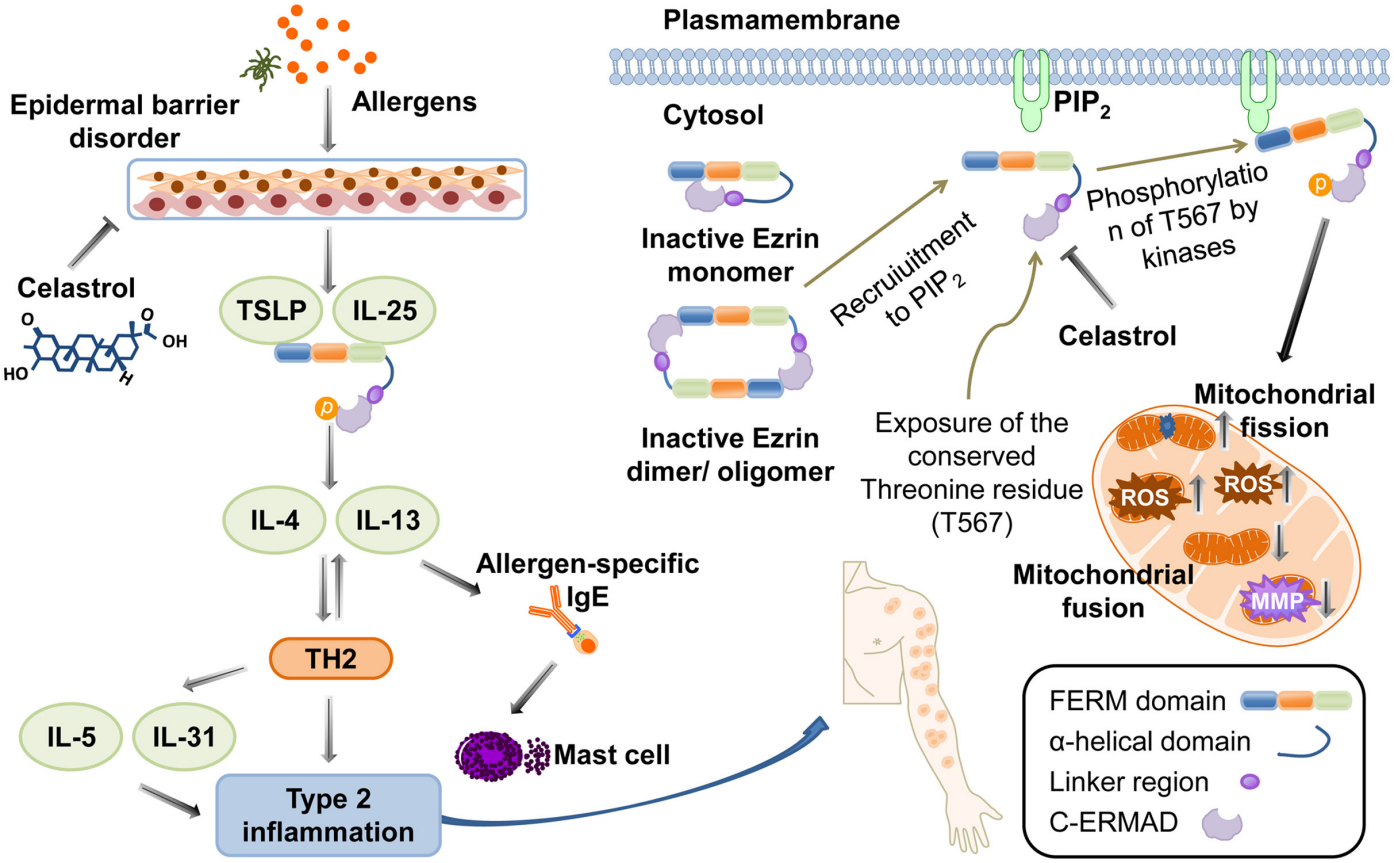
Schematic diagram illustrating the possible mechanism of celastrol in improving AD. Celastrol can inhibit the release of alarm protein TSLP, IL‐25 and Ezrin induced by allergens in keratinocytes, and inhibit the initiation of immune cascade reaction, thereby inhibiting Th2 type inflammatory response induced by IL‐4 and IL‐13 release. Celastrol can also regulate the phosphorylation of Ezrin at the T567 site, thereby mediating mitochondrial fission and fusion, ROS, mtROS and MMP generation.

In conclusion, our findings reveal that celastrol has therapeutic effects in our AD mouse model. In AD, where the integrity of the skin barrier is compromised, allergens such as mite dust, pollen and microorganisms can penetrate and induce the release of TSLP, IL‐25 and the phosphorylation of Ezrin (Figure 8). Ezrin enhances Th2‐type inflammation and induces IgE production by B cells through IL‐4/IL‐13 signalling and promotes the degranulation of mast cells. The dysregulation of Th2‐type immune responses further increases the production of IL‐4, IL‐5, IL‐13 and IL‐31 and initiates the inflammatory cascade in AD. In addition, inactive Ezrin monomers or dimers in the cytoplasm are translocated to the membrane by PIP2 (phosphatidylinositol 4,5‐bisphosphate), where they expose the phosphorylation site of the carboxyl‐terminal ERM‐associated domain at the threonine residue (T567) for Ezrin phosphorylation. This subsequently promotes mitochondrial fission, mtROS and ROS production and inhibits mitochondrial fission and MMP generation. Therefore, celastrol may serve as a natural drug for the treatment of AD, and its pharmacological effects may be attributed to the regulation of mitochondrial fission and fusion by Ezrin.

## AUTHOR CONTRIBUTIONS

**Dandan Wang:** Data curation (lead); formal analysis (lead); investigation (lead); methodology (lead); software (lead); validation (equal); visualization (lead); writing – original draft (lead). **Shan Jin:** Data curation (equal); formal analysis (equal); funding acquisition (equal); investigation (equal); methodology (equal); software (equal); writing – original draft (supporting). **Hanye Liu:** Formal analysis (equal); investigation (equal); methodology (equal). **Xinyi Song:** Formal analysis (supporting); investigation (equal); methodology (supporting). **Hongyu Jin:** Formal analysis (supporting); investigation (equal); methodology (supporting). **Yilan Song:** Formal analysis (supporting); investigation (supporting); methodology (supporting). **Hongwei Zhao:** Formal analysis (supporting); investigation (supporting); methodology (supporting). **Liangchang Li:** Conceptualization (equal); formal analysis (supporting); funding acquisition (equal); investigation (supporting); writing – review and editing (supporting). **Guanghai Yan:** Conceptualization (equal); funding acquisition (equal); resources (lead); supervision (equal); validation (equal); writing – review and editing (lead).

## CONFLICT OF INTEREST STATEMENT

The authors declare no conflicts of interest.

## Supporting information


Figure S1.

Figure S2.

## Data Availability

The datasets used and/or analysed during the current study are available from the corresponding author on reasonable request.
