# Supplementary figures and images for: Celastrol alleviates atopic dermatitis by regulating Ezrin‐mediated mitochondrial fission and fusion
Source: J Cell Mol Med. 2024 Jul 22;28(14):e18375. doi: 10.1111/jcmm.18375 (PMC11263467; doi:10.1111/jcmm.18375)

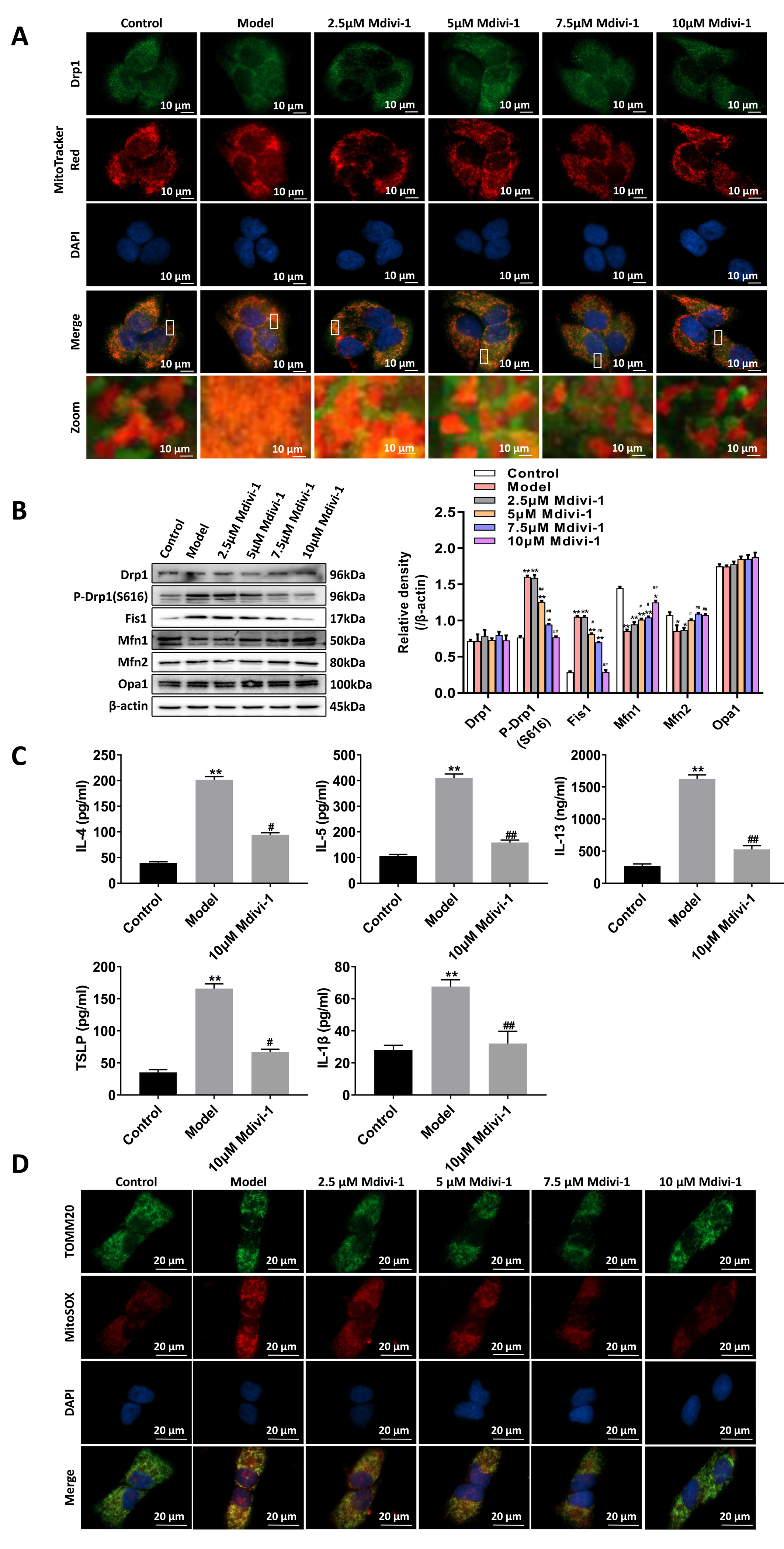

Supplement: Supplementary file 1 — Figure S1. [file JCMM-28-e18375-s001.tif]

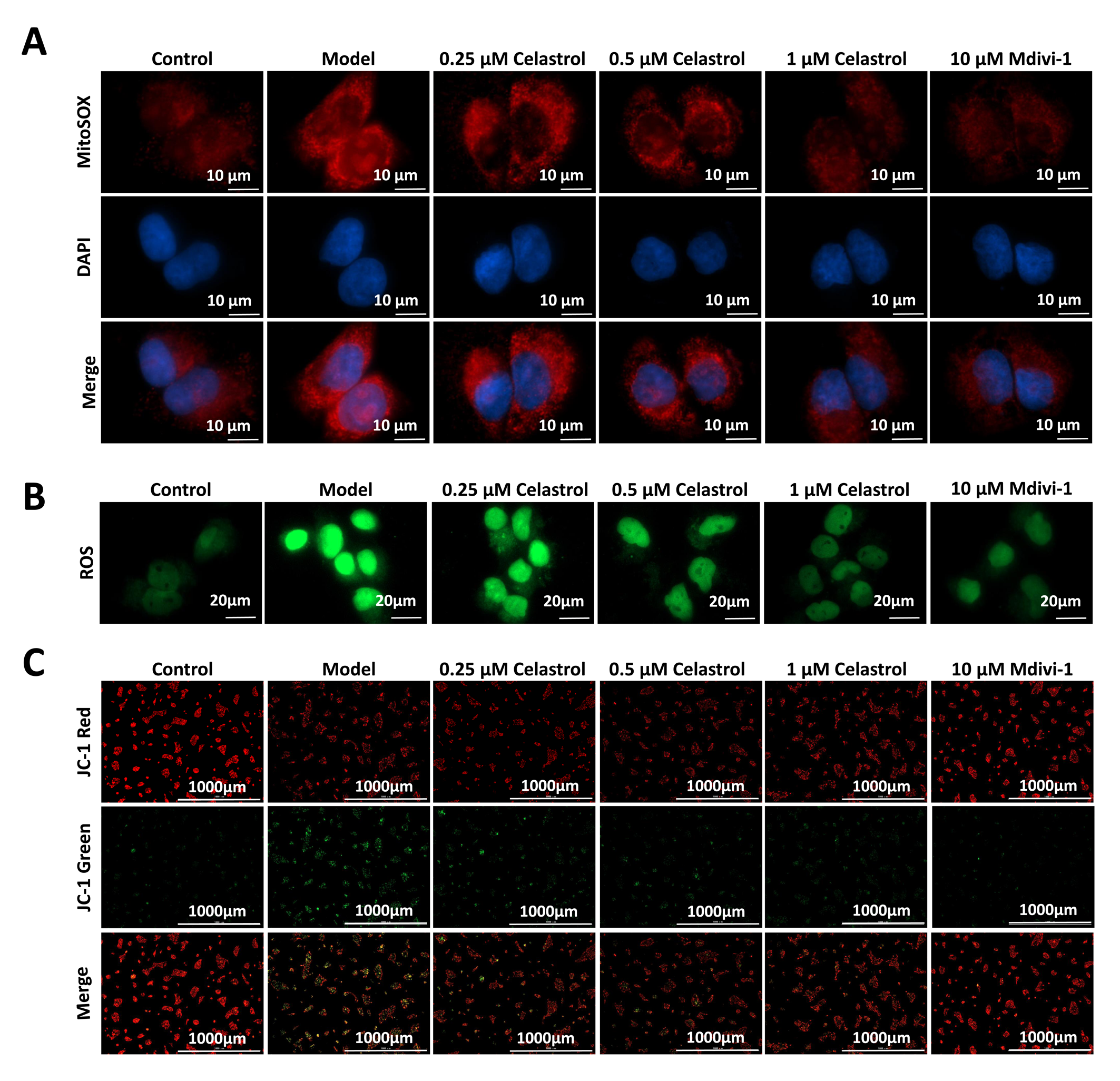

Supplement: Supplementary file 2 — Figure S2. [file JCMM-28-e18375-s002.tif]
